# The synergy of germline C634Y and V292M *RET* mutations in a northern Chinese family with multiple endocrine neoplasia type 2A

**DOI:** 10.1111/jcmm.15922

**Published:** 2020-09-29

**Authors:** Zheng Yang, Xinmeng Qi, Neil Gross, Xiujuan Kou, Yunlong Bai, Yaru Feng, Bochun Wang, Mark E. Zafereo, Guojun Li, Chuanzheng Sun, Huihui Li, Xiaohong Chen, Zhigang Huang

**Affiliations:** ^1^ Department of Otolaryngology Head and Neck Surgery Key Laboratory of Otolaryngology Head and Neck Surgery (Capital Medical University) Ministry of Education Beijing Tongren Hospital Capital Medical University Beijing China; ^2^ Department of Head and Neck Surgery The University of Texas MD Anderson Cancer Center Houston Texas USA; ^3^ Department of Epidemiology The University of Texas MD Anderson Cancer Center Houston Texas USA; ^4^ Department of Head and Neck Surgery The Third Affiliated Hospital of Kunming Medical University Kunming China; ^5^ Physical Examination Center Union Hospital Tongji Medical College Huazhong University of Science and Technology Wuhan China

**Keywords:** compound mutation, hereditary medullary thyroid carcinoma, MEN2, *RET* proto‐oncogene

## Abstract

Genetic analysis for germline mutations of *RET* proto‐oncogene has provided a basis for individual management of medullary thyroid carcinoma (MTC) and pheochromocytoma. Most of compound mutations have more aggressive phenotypes than single point mutations, but the compound C634Y/V292M variant in MTC has never been reported. Thus, we retrospectively investigated synergistic effect of C634Y and V292M *RET* germline mutations in family members with multiple endocrine neoplasia type 2A. Nine of 14 family members in a northern Chinese family underwent *RET* mutation screening using next‐generation sequencing and PCR followed by direct bidirectional DNA sequencing. Clinical features of nine individuals were retrospectively carefully reviewed. In vitro, the scratch‐wound assay was used to investigate the difference between the cells carrying different mutations. We find no patients died of MTC. All 3 carriers of the V292M variant were asymptomatic and did not have biochemical or structural evidence of disease (age: 82, 62 and 58). Among 4 C634Y mutation carriers, 2 patients had elevated calcitonin with the highest (156 pg/mL) in an 87‐year‐old male. Two carriers of compound C634Y/V292M trans variant had bilateral MTC with pheochromocytoma or lymph node metastasis (age: 54 and 41 years, respectively). Further, the compound C634Y/V292M variant had a faster migration rate than either single point mutation in vitro (*P* < .05). In conclusion, the V292M RET variant could be classified as ‘likely benign’ according to ACMG (2015). The compound variant V292M/C634Y was associated with both more aggressive clinical phenotype and faster cell growth in vitro than was either single mutation.

## 1 INTRODUCTION

Multiple endocrine neoplasia type 2 (MEN2), an inherited cancer syndrome, is classified two MEN2 syndromes: MEN2A and MEN2B. Within MEN2A, there are four variants: classical MEN2A (represented by the uniform presence of MTC and the less frequent occurrence of PHEO, or HPTH, or both), MEN2A with cutaneous lichen amyloidosis(CLA), MEN2A with Hirschsprung's disease (HD) and familial medullary thyroid carcinoma (FMTC).^1^ In 1993, Mulligan, L. M. et al originally described the causal association of germline *RET* mutations and MEN2 syndrome.^2^ To date, genetic analysis of germline mutations of the *RET* proto‐oncogene has provided a basis for the individual management of MTC and PHEO.^3^, ^4^ Many authors have reported different phenotypes for compound *RET* trans/cismutations and *RET* single point mutations.^5^, ^6^, ^7^, ^8^, ^9^, ^10^, ^11^, ^12^, ^13^, ^14^ However, more data are needed to determine the effect of compound *RET* trans/cismutations, and more relevant studies of the genotypes, phenotypes and related mechanisms of this mutation are needed to guide the individual assessment and treatment of MEN2.

In 2010, Castellone et al^3^ first described a germline V292M mutation in the extracellular region of *RET*. V292M/RET displayed detectable phosphotyrosine reactivity, but the activity of V292M was higher than that of wild‐type *RET* and lower than that of the potent C634R mutation and less potent V804M mutation. Since that time, the literature contains no reports concerning the V292M point mutation except for the description of the p.C634Y/V292M/R67H/R982C and p.V292M/R67H/R982C mutations of *RET* by Qi et al^5^ in 2011. Their results suggested that the p.C634Y/V292M/R67H/R982C transmutation of *RET* exhibited a more aggressive clinical phenotype than did the p.C634Y or p.V292M/R67H/R982C cismutation. However, they could not confirm the isolated effect of the V292M mutation since it was accompanied by the mutation of R67H/R982C.

Mutations for cysteine in extracellular domain 634 have been reported to occur in MEN2A.^15^, ^16^ The most common mutation in codon 634 is the amino acid Cys‐Arg(C634R), while the Cys‐Tyr(C634Y) mutation being less common and less aggressive than the former one.^17^ Along with all the mutations emerged in codon 634, the C634Y *RET* mutation is classified as ‘high risk’ according to the 2015 ATA guideline. Sanchez et al (1999) investigated clinical data of 49 Spanish MEN 2A families and discovered the C634Y RET mutation was positive in 73% of the families.^18^ Moreover, compound mutations including C634Y such as C634Y/Y791F, C634Y/D707E have been reported to be associated with MEN2A kindreds.^19^, ^20^

Herein, we are the first to describe a compound C634Y/V292M transmutation of *RET*, occurring in a northern Chinese family. The compound mutation was associated with a more aggressive clinical phenotype than was either the C634Y or V292M single point mutation, demonstrating the synergistic effect of compound non‐synonymous germline *RET* mutations.

## 2 MATERIALS AND METHODS

### 2.1 Study patient description

**Figure 1:**
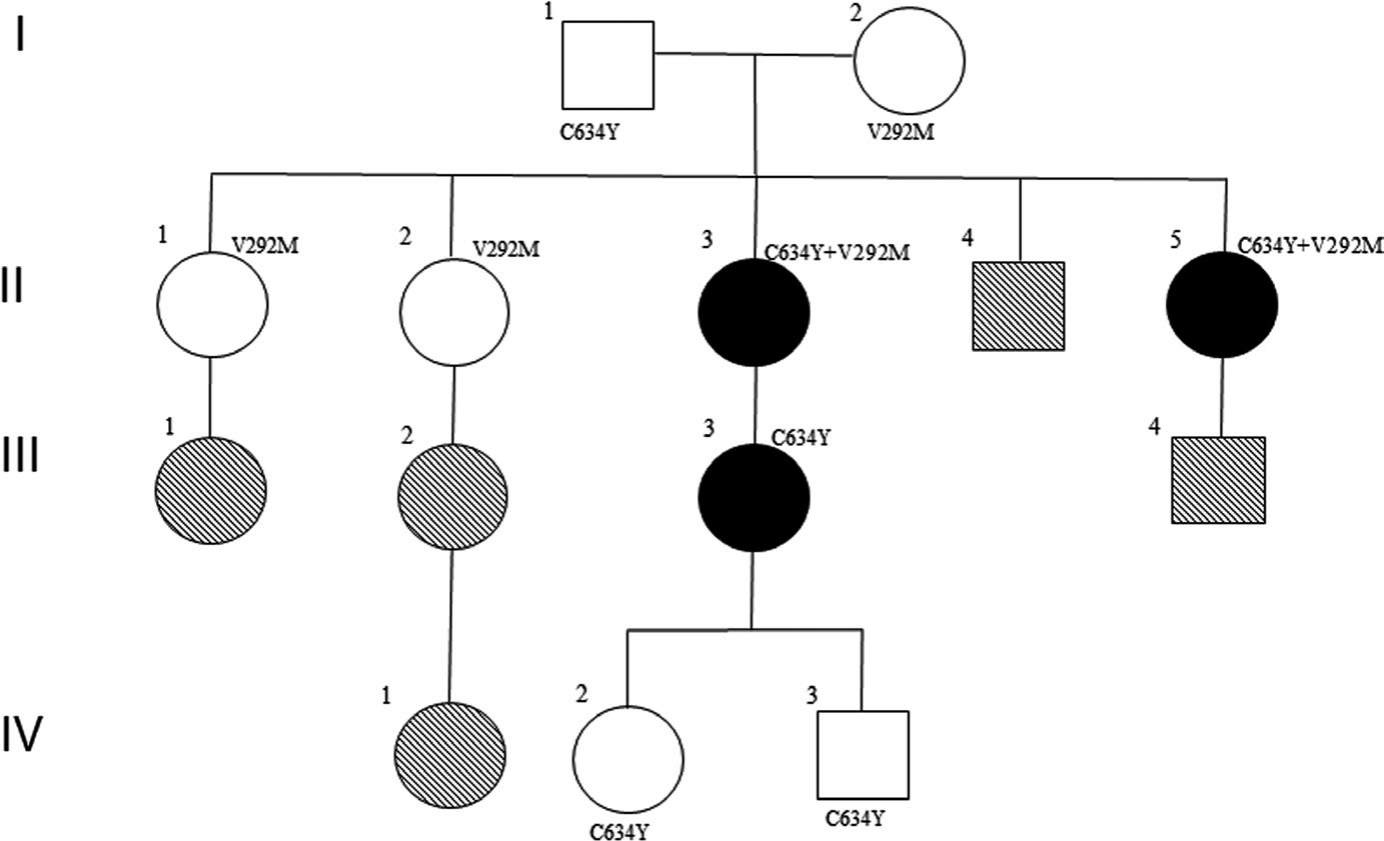
Features of the C634Y and V292M *RET* mutations among the 14 family members. Squares and circles represent the male and female family members, respectively. 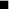 and 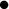 indicate positive for MTC and *RET* mutation; 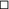 and 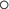 indicate negative for MTC; 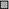 and 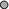 indicate not tested

The pedigree of the 14 members of the family is shown in Figure 1. Three of the participants (designated II‐3, II‐5 and III‐3) had histopathologically confirmed MTC. Nine family members underwent testing using *RET* full‐exon next‐generation sequencing and polymerase chain reaction followed by direct bidirectional DNA sequencing (MyGenostics, Beijing). The following were studied for these nine individuals: clinical and diagnostic data (age, sex and clinical features), serum calcitonin (Ct) level (normal range: 0‐11 pg/mL in females, 0‐18 pg/mL in males), carcinoembryonic antigen (CEA) level (normal range: 0‐5 ng/mL), parathyroid hormone, and Doppler ultrasound and computed tomography images including abdominal region.

### 2.2 Plasmid construction

An in vitro study was performed to characterize potentially distinct phenotypes that varied by genotype. Wild‐type complementary DNA for *RET* was obtained via gene synthesis. Point mutations of *RET* were generated using quick‐change mutagenesis. The open reading frames of wild‐type *RET* and the *RET* mutations were cloned into lentiviral vectors with puromycin resistance (GeneWay, Technology Co., Ltd.). The point mutations of *RET* were V292M c.874 G> A and C634Y c .1901 G> A.

### 2.3 Lentiviral packaging

HEK293T cells were seeded into 10‐cm culture dishes, and transfection of them with lentiviral vectors was performed with Lipofectamine 2000 (Invitrogen) when the cells were 90% confluent. Briefly, each dish was transfected with 5 μg of a lentiviral vector containing the target complementary DNA, 3 μg of a helper plasmid containing Gag/Pol/Rev (Helper 1), and 2 μg of a helper plasmid containing a VSVG envelope. Supernatants were collected 24 and 48 hours after transfection. The viral vectors were then concentrated via ultracentrifugation (Optima L‐90K, type 50.2 rotor, 80 000 *g*, 3 hours; Beckman Coulter).

### 2.4 Stable cell lines

NIH3T3 cells were cultured in high‐glucose Dulbecco's modified Eagle's medium containing 10% foetal bovine serum (HyClone Laboratories). Cells were changed to a fresh medium containing 5 μg/mL hexadimethrine bromide (polybrene) and infected with a lentivirus (multiplicity of infection, 50). Cells were then switched to a fresh culture medium. Puromycin (2 μg/mL) was added to the medium 48 hours after infection. Next, the cells were cultured in medium with 2 μg/mL puromycin and regularly changed medium. After several passages, the stable cells were used for subsequent studies.

### 2.5 Scratch‐wound assay

Obtained stable cell lines were seeded in a 24‐well plate (~30 000 cells/well). Twenty‐four hours later, the cell layer was scratched with a 200‐μL pipette tip. The width of the wound was about 200 μm. Cells were then washed once with phosphate‐buffered saline and changed to a fresh culture medium. Three spots on the bottom of the plate were labelled and then the wound area was photographed and calculated at the time of 0, 12 and 24 hours after the scratch. The cell growth rate was calculated as the percentage of newly extended area to the initial wound area.

### 2.6 Western blot

Stable cell lines overexpressing *RET*‐wt‐Flag, *RET*‐V292M‐Flag, *RET*‐C634Y‐Flag, *RET*‐V292M+C634Y‐Flag and *RET*‐V292M/C634Y‐Flag were seeded in a six‐well plate. Forty‐eight hours later, cells were washed with 1x phosphate‐buffered saline twice and lysed in 200 μL of RIPA buffer. Lysates were cleared of cellular debris via centrifugation, subjected to 10% sodium dodecyl sulphate‐polyacrylamide gel electrophoresis and wet‐transferred to nitrocellulose membranes. Next, the nitrocellulose membranes were blocked for 1 hour with 5% no‐nfat milk and incubated overnight with a FLAG/β‐actin antibody (1:1000; Affinity Biosciences), washed three times with Tris‐buffered saline with Tween‐20 and incubated for 1 hour with a goat anti‐mouse horseradish peroxidase‐conjugated secondary antibody (Sangon Biotech).

### 2.7 Statistical analyses

Data presented as mean ± SD were analysed using SPSS version 19.0 (SPSS Inc). The differences between groups were performed with *t* test or one‐way ANOVA. A value of *P* < .05 was defined as statistically significant Results. All in vitro assays were triplicated.

## 3 RESULTS

### 3.1 Characterization and distributions of RET mutations among 14 family members

**Figure 2:**
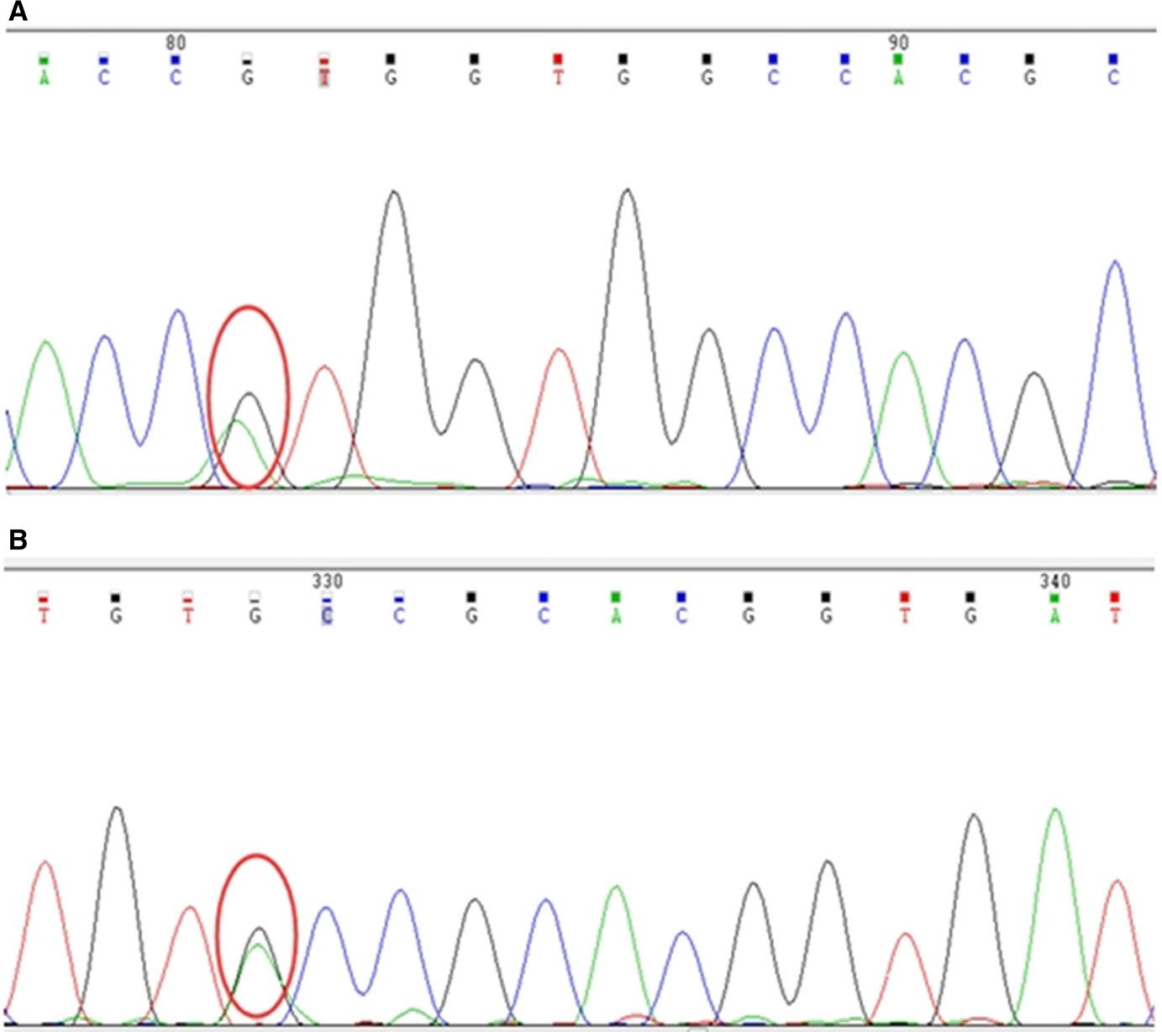
The *RET* mutations in the family members studied. A, V292M mutation: base A was replaced by base G (complementary chain T>C) at position chr10‐43601830. B, C634Y mutation: base G was replaced by base A (complementary chain C>T) at position chr10‐43609949

We purified genomic DNA from participant II‐5’s peripheral blood and tested it using *RET* full‐exon next‐generation sequencing. In total, we identified two *RET* missense mutations in participant II‐5. They are c.1901G>A (p.C634Y) at position chr10‐43609949 and *RET* mutation c.874G>A (p. V292M) at position chr10‐43601830 in exon 5 (Figure 2). We tested the genomic DNA from participant II‐5’s parents using the same method and found the p.C634Y and p. V292M mutations in her father and mother, respectively. We then performed polymerase chain reaction followed by direct bidirectional DNA sequencing for other available family members to examine for C634Y and V292M mutations. We found the compound C634Y/V292M mutation in participant II‐3 and II‐5; V292M mutation in participants I‐2, II‐1 and II‐2; and the single mutation of C634Y in participants I‐1, III‐3, IV‐2 and IV‐3.

### 3.2 Clinical features of the family members

Table 1 demonstrates clinical characteristics of 9 *RET*‐positive family members. Participant II‐5 was 41 years old and had an abnormally elevated CEA level (39 ng/mL). She underwent a total thyroidectomy and central compartment lymphadenectomy, with pathology demonstrating a 2 cm left and 0.8 cm right MTC with lymphatic metastasis in all 7 lymph nodes. She also had a nodule in her adrenal gland, but it was not clearly a pheochromocytoma. Her postoperative Ct and CEA levels were in the normal range. Her older sister, participant II‐3, underwent total thyroidectomy for MTC and right adrenal pheochromocytoma resection at the age of 54 years. Her daughter, grandson and granddaughter were all C634Y mutation carriers. Her daughter had multiple thyroid nodules and an abnormally high Ct level (119 pg/mL), so she underwent surgery for the MTC at the age of 32 years. She had no lymph node involvement. Regarding this participant's two children, her daughter (IV‐2) had thyroid micronodules (<2 mm in diameter) and an abnormally high Ct level (16 pg/mL), whereas her son (IV‐3) also had a 2 mm thyroid micronodule but a normal Ct level. However, their parents refused prophylactic thyroidectomy for their children. Participants II‐1 and II‐2 were carriers of the V292M mutation only and had normal Ct and CEA levels. Participant II‐2 had only a small thyroid micronodule (diameter, 2 mm). Participant II‐1 underwent surgery for a symptomatic multinodular thyroid goitre, with pathology demonstrating a benign multinodular goitre without MTC. The mother (I‐2), who carried the same mutation, had normal basal serum Ct and CEA levels and hypertension. The father (I‐1), who was a C634Y point mutation carrier, had relatively and slightly high levels of Ct (156 pg/mL) and CEA (9 ng/mL). Because of their advanced age, I‐1 and I‐2 declined further evaluation and imaging.

**Table 1:** Clinical characteristics for 9 RET mutation‐positive family members

Family member	Sex	Age (y)	ADM (y)	Mutation	Pre‐op Ct level (pg/mL)	Pre‐op CEA level (ng/mL)	DUS result	Histology	LN
I‐1	M	87	NA	C634Y	156	9	NA	NA	NA
I‐2	F	82	NA	V292M	9	1	NA	NA	NA
II‐1	F	62	NA	V292M	2	1	NA	Benign	NA
II‐2	F	58	NA	V292M	2	2	L, 0.2 cm (cystic)	NA	NA
II‐3	F	56	54	C634Y/V292M	NA	NA	L, 2.0 cm; R, 2.2 cm	MTC (bilateral) R‐PHEO	NA
II‐5	F	43	41	C634Y/V292M	NA	39	L, 2.0 cm; R, 0.8 cm	MTC (bilateral)	7/7
III‐3	F	32	32	C634Y	119	4	L, 1.5 cm; R, 0.6 cm	MTC (bilateral)	NA
IV‐2	F	8	NA	C634Y	16	2	R, 0.2 cm; L, 0.1 cm	NA	NA
IV‐3	M	6	NA	C634Y	7	1	L, 0.2 cm	NA	NA

Abbreviations: ADM, age at diagnosis of MTC; Pre‐op, preoperative; DUS, Doppler ultrasound; LN, lymph node; M, male; NA, not available or not applicable; F, female; L, left; R, right; Ct, serum calcitonin (normal range: 0‐11 pg/mL in females, 0‐18 pg/mL in males); CEA, carcinoembryonic antigen (normal range: 0‐5 ng/mL); ADM, age at diagnosis of MTC; Pre‐op, preoperative; DUS, Doppler ultrasound; LN, lymph node; M, male; NA, not available or not applicable; F, female; L, left; R, right; Ct, serum calcitonin (normal range: 0‐11 pg/mL in females, 0‐18 pg/mL in males); CEA, carcinoembryonic antigen (normal range: 0‐5 ng/mL).

### 3.3 *Analysis of our* in vitro*study*

**Figure 3:**
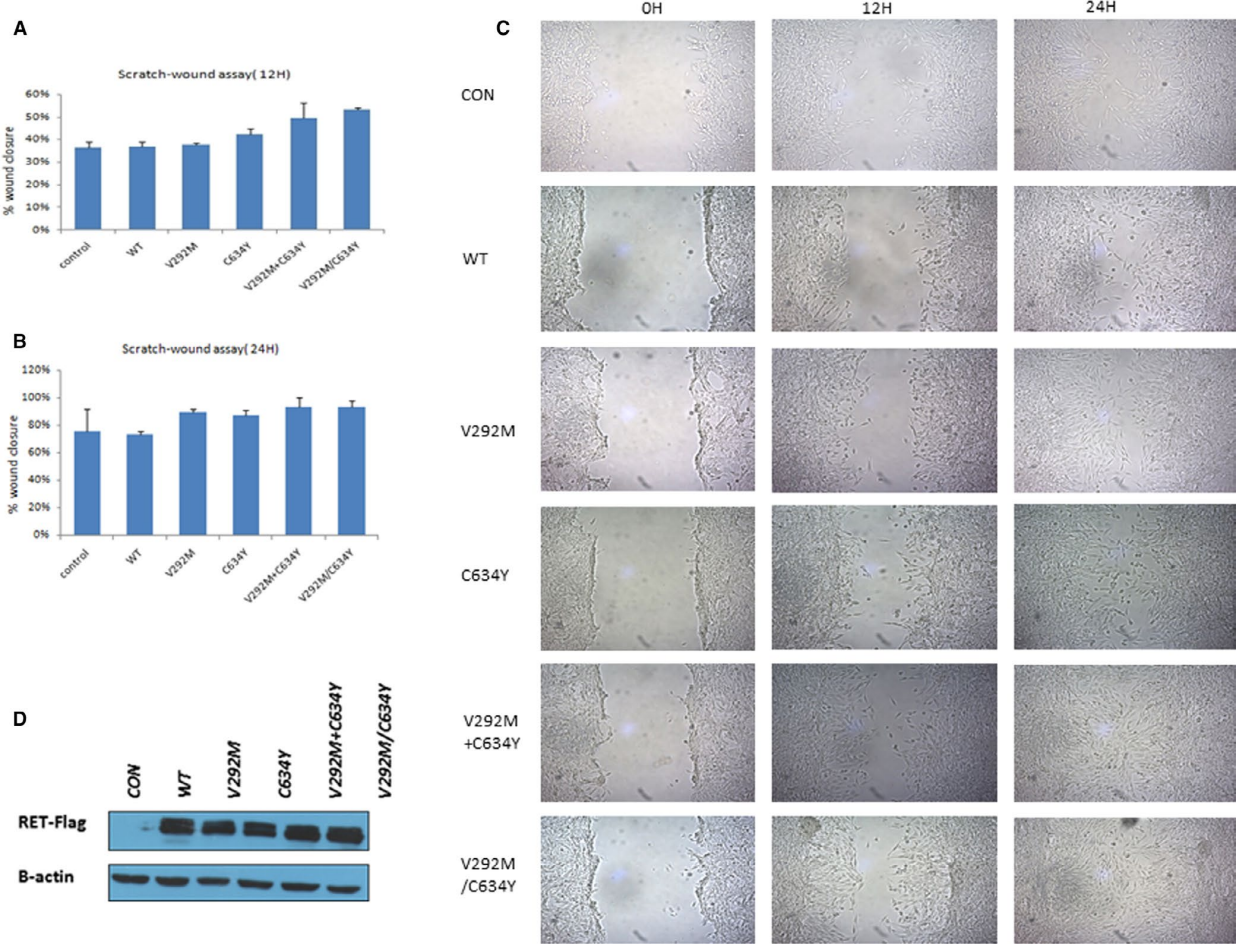
The migratory ability of the V292M and C634Y *RET* single mutations and the compound C634Y/V292M mutation according to a scratch‐wound assay. A, Scratch‐wound assay results at 12 h. B, Scratch‐wound assay results at 24 h. C634Y/V292M, transmutation; C634Y+V292M, cismutation. C, Representative images obtained at 0 and 12 and 24 h after wounding (magnification, 10×). D, Western blotting assays from cells individually transfected with V292M and C634Y *RET* single mutations (lanes 3 and 4), the V292M+C634Y mutant and the compound C634Y/V292M mutant (lanes 5 and 6). Lane 1 is an empty vector, and lane 2 represents wild‐type *RET* gene

We used the scratch‐wound assay to investigate the growth of cells harbouring the *RET* mutations described above, which are indicative of the migratory ability of the mutations. At the point of 12 hours after the wounds were created, the wound closure for control, wild‐type *RET*, V292M, C634Y, V292M+C634Y and V292M/C643Y groups were 36.41%, 36.96%, 37.65%, 42.36%, 49.32% and 53.16%, respectively. C634Y and compound variants significantly fastened cell migration compared to V292M, wild‐type *RET* and control groups (*P* < .05). And that the V292M/C643Y cells possess greater migration ability than C634Y cells had statistical significance (*P* < .01). For the 24‐hour observation, the wound closure for control, wild‐type *RET*, V292M, C634Y, V292M+C634Y and V292M/C643Y groups were 75.82%, 73.85%, 89.46%, 87.29%, 93.74% and 93.30%, respectively. Statistical significance was observed between V292M, C634Y, V292M+C634Y, V292M/C643Y groups and wild‐type *RET* cells, respectively (*P* < .01). Expression of compound mutations significantly displayed greater ability to close the wound than did those with expression of V292M for the 12‐hour group (Figure 3A‐C). We performed Western blotting of RET protein expression in these cells using an anti‐FLAG antibody. The results showed that RET protein could be expressed stably in each transfected cell line (Figure 3D).

## 4 DISCUSSION

Several compounds *RET cis* and *trans*mutations have been described in the literature. Most of them have a more aggressive phenotype than do single mutations except for p.R844L, which has an inhibitory effect on p.V804M.^11^ In the present study, we found that MEN2A was associated with the compound C634Y/V292M *RET* transmutation in several family members and that the compound mutation appears to result in a more aggressive clinical phenotype than do the single C634Y and V292M point mutations. Moreover, the clinical features of C634Y and V292M mutation carriers were different from those reported previously.

Composed of 21 exons, the proto‐oncogene *RET* is located on chromosome 10 (10q11.2) and encodes for a transmembrane receptor tyrosine kinase for members of a glial cell line.^21^, ^22^ The *RET* protein is composed of an extracellular ligand‐binding domain containing a cysteine‐rich region, a series of cadherin homology domains, a transmembrane domain and a cytoplasmic tyrosine kinase domain. The highly conserved cysteine‐rich region is important for disulphide bond formation, which is required for maintaining the native tertiary structure, allowing for receptor dimerization.^22^ In the majority of MEN2A families (more than 90%), germline mutations affect the cysteine‐rich extracellular domain by converting a cysteine into another amino acid, and this mutation determines *RET* spontaneous dimerization and activation.^3^, ^5^, ^7^ These mutations are located at codon 634 (exon 11) or codons 609, 611, 618 and 620 (exon 10), respectively. Codon 634 mutation is associated with the more severe forms of the MEN2A phenotype, and it is classified as high risk of aggressive MTC in the American Thyroid Association (ATA) guidelines.^4^

There are six different amino acid substitutions for the same cysteine on codon 634 (F/G/R/S/W/Y), and all of them display comparable transforming activity. The C634Y mutation is believed to confer lower penetrance of the MEN2A phenotype and less aggressive behaviour of MTC than does the C634R mutation.^23^, ^24^, ^25^ There is higher penetrance of MTC, phaeochromocytoma and hyperparathyroidism in patients harbouring C634R mutation. The age‐related penetrance of bilateral phaeochromocytoma in C634R is significantly higher than in other types of codon 634 mutations. Lymph node and distant metastases occurred earlier in C634R carriers than in the C634Y carriers. The C634R mutation is reported to be an independent factor for recurrent or persistent disease. In Chinese population the most frequent *RET* proto‐oncogene mutation was localized at codon 634 of exon 11, with the C634Y mutation as the most common, followed by C634R, C634W and the rarer mutations C634F, C634S and C634G.^26^, ^27^ In the present study, the 87‐year‐old C634Y carrier (I‐1) had unexpected and slightly high levels of Ct (156 pg/mL), suggesting that his MTC would be associated with relatively moderate biological behaviour and lesser aggressiveness of disease. The participants IV‐2 and IV‐3 are C634Y carriers, and according to ATA guidelines, they should have undergone prophylactic thyroidectomy before turning 5 years of age. If their mutation status had been known in childhood, and considering the relatively moderate clinical features of the C634Y mutation carriers in this family, prophylactic thyroidectomy could have been recommended to be postponed for these two individuals until 8 years old. Other studies of MEN2A patients with C634Y mutation in the Chinese population have drawn similar conclusions.^26^, ^27^

Castellone et al^3^ described an Italian MEN2A patient carrying the V292M *RET* mutation in an extracellular region who had a 4‐mm unifocal MTC and right PHEO, no metastases in four resected lymph nodes and C cell hyperplasia foci in both thyroid lobes. An in vitro study confirmed the pathogenicity of the V292M *RET* mutation, with demonstrating detectable phosphotyrosine reactivity greater than that for wild‐type *RET* but less than that for the potent C634R mutant and less potent V804M mutant. In the family members of the present study, the V292M carriers seemed to have nonpathogenic or weakly pathogenic mutations, as the basal serum Ct and CEA levels in all V292M mutation carriers were normal. Even if it is a pathogenic mutation, the phosphotyrosine reactivity would be suspected to be lower than that for p.V292M and p.V292M/R67H/R982C reported by Castellone et al^3^ and Qi et al,^5^ respectively, because of the carriers’ lack of clinical manifestations.

V292M RET variant was described in germline of Vietnamese patients in cohort of 97 cases of Southern Chinese ancestry with Hirschsprung's disease.^28^ The frequency of this rare variant seems to be higher than estimated prevalence of MEN2 when we referred to gnomAD database. Also, this variant was found in gnomAD database in variable frequencies and with higher frequency in South Asia (0.036% or 1/2774) and East Asia (0.7% or 1/142) than in other regions. There is no apparent data about this variant from Northern region of the China. In Varsome, V292M is classified as uncertain significance variant (VUS) and in ClinVar as conflicting interpretations of pathogenicity varying from benign up to VUS. In ICGC somatic, a Cancer Database, V292M was found in 2% of tumours (1/50). We also referred to the 2015 ACMG guideline and V292M variant could be considered as ‘likely benign’, due to the facts that this variant meets with the criteria of PP2, PP4, PP5, BP2 and BP6. In MEN2, we have the example of the Y791F, a RET variant initially reported as pathogenic. Recent study from Brazilian and Germany groups positioned it as a benign variant.^29^, ^30^, ^31^ This classification has been very recently reinforced for Danish study.^32^ Curiously, functional studies of Y791F, as occur with V292M, suggest pathogenicity to this variant. Toledo et al^7^ discussed in their paper that Y791F may represent a benign RET variant with potential to modulate phenotype considering to previous report of them documenting high penetrance of PHEO in cases harbouring C634Y+Y791F cis association.^20^ Therefore, it is possible that V292 represents more a VUS or same a MEN2 phenotype modulating benign variant.

The clinical features of the C634Y mutation carriers in this study seemed to be relatively moderate. However, we could not confirm the exact role of the C634Y mutation in these patients because of limited case numbers. The formation of a tumour is well known to be the result of synergistic or sequential external environmental factors and genetic carcinogenic factors. *RET* point mutations may play a dominant role in the development of hereditary MEN2, but we still know little about other specific influencing factors that explain the various phenotypes within families. For example, the possibility that polymorphisms may act as low susceptibility factors or as modifiers of a specific disease which have been reported. Increasing studies are pointing to *RET* single‐nucleotide polymorphisms which are believed to be genetic modifiers in individual development of MTC.^20^, ^33^, ^34^, ^35^, ^36^

In summary, the V292M variant did not have significant clinical impact on family members in this cohort, and the clinical features of the C634Y mutation carriers seemed to be relatively moderate. The more aggressive clinical phenotype in the compound mutation than in the single mutation carriers, and the results of the in vitro study, demonstrated the synergistic effect of the two mutations when they occurred simultaneously. Furthermore, cases with compound mutation with potential to modify phenotypes should be managed according to the recommendations in published guidelines. And the various phenotypes in the family members demonstrated the importance of individual assessment and treatment of MEN2A in the future. A limitation of this study is that the sample size was relatively small, and this could lead to a biased finding; and at the same time, we could not exclude other factors rather than *RET* mutations which may influence the specific clinical phenotypes. Also, further in vitro studies are necessary to explore the synergistic effect mechanism of the compound C634Y/V292M genotype.

## 5 CONCLUSION

We are the first to describe the compound C634Y/V292M *RET* trans association. Additionally, the compound C634Y/V292M mutation resulted in an apparently more aggressive phenotype than did either the C634Y or V292M single point variant. Treatment of MEN2A should be individualized based on particular associated mutations and any available family history about the behaviour of family members with the same mutations, while research should continue to elucidate the optimal therapeutic schedule for carriers of each mutation.

## CONFLICT OF INTEREST

The authors declare no conflict of interest.

## AUTHOR CONTRIBUTIONS

Zheng Yang: Data analysis and manuscript preparation. Xinmeng Qi: Performing of the assays and manuscript preparation. Neil Gross: Review of the manuscript. Xiujuan Kou: Patient communication and collection of clinical data. Yunlong Bai: Performing of the assays. Yaru Feng: Performing of the analysis. Bochun Wang: Performing of the assays. Mark E Zafereo: Review of the manuscript. Guojun Li, Huihui Li and Chuanzheng Sun: Reagents, materials and analysis tools. Xiaohong Chen: Supervising of the whole study. Zhigang Huang: Conception of the study.

## INFORMED CONSENT

This study was approved by the Ethics Committee (TRECKY2014‐011) of Beijing Tong Ren Hospital. All participants and/or their legal guardians provided written informed consent for participation in the present study.

## Data Availability

The data that support the findings of this study are available on request from the corresponding author. The data are not publicly available due to privacy or ethical restrictions.
